# Atomic Layer Deposition of ZnO and ZnO/Cu Coatings for Fresh Food Packaging Application

**DOI:** 10.3390/polym18060751

**Published:** 2026-03-19

**Authors:** Adriana Lordi, Regina Del Sole, Fabio Palumbo, Alberto Perrotta, Francesco Fracassi, Marianna Roggio, Antonella Milella, Amalia Conte, Matteo Alessandro Del Nobile

**Affiliations:** 1 Dipartimento di Scienze Sociali, Università di Foggia, 71122 Foggia, Italy; adriana.lordi@unifg.it; 2 Dipartimento di Chimica, Università degli Studi di Bari “Aldo Moro”, 70125 Bari, Italy; regina.delsole@uniba.it (R.D.S.); francesco.fracassi@uniba.it (F.F.); marianna.roggio@uniba.it (M.R.); 3 Istituto di Nanotecnologia, Consiglio Nazionale delle Ricerche, S.S. Bari, c/o Dipartimento di Chimica, Università degli Studi di Bari “Aldo Moro”, 70125 Bari, Italy; fabio.palumbo@cnr.it (F.P.); alberto.perrotta@cnr.it (A.P.); 4 Dipartimento di Studi Umanistici, Lettere, Beni Culturali, Scienze della Formazione, Università di Foggia, 71121 Foggia, Italy; 5 Dipartimento di Economia, Management e Territorio, Università di Foggia, 71122 Foggia, Italy; matteo.delnobile@unifg.it

**Keywords:** active packaging, ALD, zinc oxide, copper, shelf life

## Abstract

Active antimicrobial films based on polyethylene terephthalate (PET) were developed through atomic layer deposition (ALD) and plasma sputtering to obtain ZnO (≈15 nm) and ZnO/Cu (≈18 nm) coatings. Surface characterization by X-ray photoelectron spectroscopy confirmed zinc in ZnO form and copper as Cu_2_O/CuO, while mass spectrometry quantified approximately 10 µg/cm^2^ of Zn in both samples and about 130 ng/cm^2^ of Cu in the ZnO/Cu films. The antimicrobial performance of the coatings was evaluated on burrata cheese and turkey fillets stored under refrigeration, assessing microbial growth and sensory quality over time. The films exhibited different effects depending on food type and the initial contamination levels. On burrata cheese, PET-ZnO moderately extended the shelf life by inhibiting *Pseudomonas* spp., while PET-ZnO/Cu further enhanced preservation. Cheese packaged with PET-ZnO/Cu remained acceptable for over 21 days compared to 19–20 days for the controls. More pronounced effects were observed in turkey fillets, characterized by a higher initial contamination. In control samples, *Staphylococcus* spp. rapidly proliferated, leading to spoilage within one day. Both active films significantly delayed microbial growth and sensory decay, with PET-ZnO/Cu providing the best performance, extending acceptability beyond two days compared to less than one day for the controls.

## 1. Introduction

Active packaging represents an innovative approach for food preservation since it actively interacts with food products to preserve quality and extend the shelf life [1,2]. These films can scavenge oxygen, moisture, or ethylene, thereby slowing down oxidation, microbial spoilage, and food senescence [3]. Alternatively, they can release active agents such as natural antioxidants or antimicrobial compounds that can directly prevent food deterioration [4]. For instance, inorganic nanomaterials are renowned for their antimicrobial activity and for offering the advantage of being safer and more stable compared to organic ones, thus, they can be exploited for the fabrication of active packaging [5]. Metal and metal oxide nanoparticles have been demonstrated to possess remarkable antimicrobial efficiency, therefore, they play a central role in the development of innovative active food packaging. Traditionally, inorganic nanoparticles are incorporated via wet chemistry methods into polymer matrices to cast or spray films with more appreciable characteristics for food packaging material production [6,7].

Inorganic functional coatings can also be deposited using vapor-phase techniques such as physical vapor deposition (PVD) and chemical vapor deposition (CVD), which have been widely employed to produce barrier and antimicrobial films on polymeric substrates [8,9,10]. PVD techniques, including sputtering and evaporation, allow for the deposition of metals and metal oxides with good adhesion and relatively high deposition rates. However, the line-of-sight nature of PVD processes may limit coating conformality on complex or porous surfaces. Conversely, CVD methods enable the formation of uniform coatings through gas-phase chemical reactions, but they often require relatively high temperatures that may not be compatible with thermally sensitive polymer substrates commonly used in food packaging. Atomic layer deposition (ALD) can be considered as a particular class of CVD based on sequential, self-limiting surface reactions. This mechanism allows for precise thickness control at the atomic scale and excellent conformality even on complex or deformable substrates, while operating at relatively low temperatures [11]. These characteristics make ALD particularly attractive for the fabrication of functional coatings on polymeric materials used in food packaging applications. Indeed, ALD is particularly attractive for the preparation of antimicrobial thin films as, through precise thickness control, it can address good activity with a minimal amount of active material. Moreover, these processes guarantee an extreme adhesion of the coating to the polymeric material [12,13], thus limiting the migration of particles inside food and avoiding health issues to consumers related to the cytotoxicity limits of the deposited materials [14]. Effective antibacterial single layers such as TiO_2_, ZnO, Fe_2_O_3_, MgO and ZrO_2_ thin films have been successfully deposited via ALD [15]. On the other hand, RF sputtering is a valuable technique for the deposition of metal nanoparticles (e.g., Ag and Cu) and coatings with antibacterial properties [16].

ZnO coatings are a particularly interesting choice for the development of active packaging as they combine the gas barrier properties with a remarkable antibacterial activity [17]. This latter is generally due to the release of Zn^2+^ ions into an aqueous environment, which are transported across the cellular membrane to the cytoplasm, where they can interact with the active protease, reducing the ATP levels in bacteria. Moreover, even the sharp morphology of nanostructured ZnO can induce cell membrane rupture during adhesion. Finally, under irradiation, the ZnO antibacterial activity is further enhanced: due to its band gap around 3.3 eV, ZnO can absorb light in the UV wavelength range, generating reactive oxygen species (ROS) in water, which are mainly responsible for cell membrane damage by induced oxidative stress [18]. However, similarly to other n-type semiconductors, ZnO photoactivity is affected by a fast electron-hole recombination rate. To even further enhance its photoactivity, ZnO is often coupled in nanocomposites with metal nanoparticles that form a heterojunction with ZnO, limiting the electron-hole recombination rate [19]. In addition, metal nanoparticles can release metal ions, which themselves possess broad-spectrum antibacterial properties. This is the case of copper, which is often used alone [20] and in combination with metal oxides for functional antibacterial material production [21].

In the food packaging field, extending the shelf life of highly perishable foods remains a major challenge, especially for products with high moisture content and nutrient-rich compositions that favor microbial growth. Fresh turkey fillets are highly perishable due to a nutrient-rich composition, favorable for microbial growth, with a shelf life of a few days under refrigeration. Burrata is a high-moisture, high-fat, fresh pasta filata cheese with a shelf life limited to a few days under refrigeration due to the rapid growth of spoilage microorganisms such as *Pseudomonas* spp. and *Enterobacteriaceae* [22].

This work presents, for the first time, to the best of the authors’ knowledge, a thorough investigation of the effect of ZnO and ZnO/Cu coatings, deposited combining thermal ALD and RF sputtering, on the shelf life of turkey fillets and burrata cheese.

## 2. Materials and Methods

### 2.1. ZnO and ZnO/Cu Film Preparation

Commercial PET film (Biaxially Oriented, 100 µm thickness, Goodfellow, Huntingdon, UK) was cut in squares (3 cm × 3 cm) and cleaned with ethanol in ultrasonic bath (CP102, Ceia spa, Arezzo, Italy) at 130 W for removing surface contaminants. The substrates were then dried with a nitrogen gun before the deposition of the active layer by ALD and RF sputtering. A custom-built ALD reactor was used for both the deposition of ZnO and ZnO/Cu coatings. The core of the reactor was a cylindrical stainless steel vacuum chamber evacuated by a turbomolecular (Pfeiffer Vacuum TC600, Asslar, Germany)/rotary system (Pfeiffer Vacuum DUO 20 MC, Asslar, Germany) and equipped with a resistive heating plate, where the PET substrates were placed along with slices of double polished p-doped Si wafer (675 µm thickness, 1.5 cm × 1.5 cm, MicroChemicals, Ulm, Germany). For the deposition of ZnO coatings, diethyl zinc (DEZ; purity > 95%, Dockweiler Chemicals GmbH, Marburg, Germany) was used as the metalorganic precursor, and double distilled water was used as the co-precursor for the ALD deposition of ZnO. Argon (purity 99.995%, Air Liquide, Paris, France) was added as a buffer gas for the removal of by-products and unreacted precursors from the chamber, and the flow rate was set at 50 sccm by an electronic mass flow controller (MKS Instruments, Andover, MA, USA). The pressure in the chamber was monitored by a capacitive gauge (Pfeiffer Vacuum CMR-263, Asslar, Germany) and set at a base value of 90 mTorr. DEZ was pulsed into the reactor by means of an ALD valve (Fitok ALD Series, New Taipei City, Taiwan) while Ar and water were admitted by pneumatic valves (Swagelok, Solon, OH, USA). To avoid condensation, the water delivery line was heated to 50 °C. The whole process was carried out at a reactor temperature of 80 °C, while the substrate holder was heated up to 100 °C. The optimized thermal ALD recipe consisted of the sequence <DEZ pulse/Ar purge/H_2_O pulse/Ar purge> with pulse times <0.15/25/1/25> in seconds. The deposition was carried out for 75 cycles.

For ZnO/Cu coatings, the above-described ALD process was followed by a plasma sputter deposition, which was carried out in the same chamber. For this aim, the ALD reactor was integrated with an RF magnetron sputtering source (ST30, AJA International, Inc., Hingham, MA, USA) bearing a 3″ copper target (purity 99.999%). The top RF electrode was connected to a 13.56 MHz power supply (Cesar 1310, Dressler GmbH, Stolberg-Vicht, Germany) through an automatic impedance matching unit (Variomatch, Dressler GmbH, Stolberg-Vicht, Germany). Reactor and plate temperature, Ar flow rate, and base pressure were kept constant, passing from ALD to sputtering mode. RF power and deposition time were set to 20 W and 34 s, respectively, to obtain the desired thickness.

### 2.2. Film Characterization

The ZnO and ZnO/Cu films deposited on PET (PET-ZnO and PET-ZnO/Cu, respectively) were thoroughly characterized from the morphological and chemical point of view. The thickness of the ZnO and ZnO/Cu coatings deposited onto control Si slices was measured by spectroscopic ellipsometry (SE) (J. A. Woollam Co., Inc., Lincoln, NE, USA, M-2000UI EC-400). The spectra were acquired at three different incidence angles (60°, 65°, and 70°) in a wavelength range of 250–1700 nm. The spectra were modeled with the software Complete EASE (Version 4.92, J. A. Woollam Co., Inc.). A three-layer model, considering the Si substrate with backside reflection correction, a SiO_2_ native layer with an average thickness of 1.80 nm, and a ZnO layer was used in the case of the ZnO samples. The ZnO layer was modeled with a Cody Lorentz dispersion function that also took into account the absorption at energies below the bandgap. For ZnO/Cu coatings, a fourth effective medium approximation (EMA) layer, considering the presence of sputtered Cu partially oxidized to CuO, was added to the model.

Surface morphology was evaluated using an XE-70 atomic force microscope (AFM, Park Systems, Suwon, Republic of Korea) operating in non-contact mode using PPP-NCHR probes from Nanosensors (Neuchâtel, Switzerland) with a resonance frequency of 330 kHz.

The surface chemical composition of the samples was investigated by X-ray photoelectron spectroscopy (XPS) analyses with a PHI 5000 Versa Probe II spectrometer (Physical Electronics, Chanhassen, MN, USA) equipped with a monochromatic Al Kα X-ray source (1486.6 eV), operated at 15 kV and 24.8 W, with a spot size of 100 µm. Survey (0–1200 eV) and high-resolution spectra (C1s, O1s, Zn2p_3/2_, ZnLMM, Cu2p and CuLMM) were recorded in FAT (Fixed Analyzer Transmission) mode at a pass energy of 117.40 eV and 29.35 eV, respectively. Surface charging was compensated using a dual beam charge neutralization system, with a flux of low energy electrons (∼1 eV) combined with very low energy positive Ar^+^ ions (10 eV). Binding energy scale was referenced to the C1s core level photoemission line at 284.8 eV. All spectra were collected at an angle of 45° with respect to the sample surface. Curve fitting of the high-resolution spectra was carried out with PHI MultiPak V.9.5 data processing software (Physical Electronics) using a Shirley background and Gaussian/Lorentzian peak shape.

The amount of Zn and Cu deposited in the ZnO and ZnO/Cu coatings deposited on Si was determined by inductively coupled plasma-mass spectrometry (ICP-MS) analysis (Thermo, Waltham, MA, USA, ICAP RQ) in accordance in accordance with standard method [23]. Samples for ICP-MS analysis were prepared as follows. A Si slice coated with the deposited thin film (1.5 cm × 1.5 cm) was digested using 2 mL of HNO_3_ (69% *w*/*w*, PanReac, AppliChem, Darmstadt, Germany) and 3 mL of HCl (37% *w*/*w*, J. T. Baker, Phillipsburg, NJ, USA), at 70 °C until complete evaporation. The resulting residue was recovered using a solution of HNO_3_ (2% *w*/*w*) and then filtered with a PTFE hydrophilic filter (pore size of 1 μm).

The leaching in water of the films deposited on PET was evaluated by keeping the samples in 50 mL double distilled water (DDW) for a total of 21 days, to account for the time considered for the microbiological test. Two aliquots of DDW of 1 mL each were withdrawn after 4 and 21 days. The amounts of Zn and Cu released in these timespans were quantified by ICP-MS (ICP MS Agilent 7850, Santa Clara, CA, USA) in accordance with [23], after the addition of 10 mL HNO_3_ (69% *w*/*w*, PanReac, AppliChem).

### 2.3. Burrata and Turkey Fillet Packaging

To test the efficacy of the prepared active films, two fresh products were selected: burrata cheese and turkey fillet. These foods were selected because they are highly perishable products with a relatively short shelf life, making them an appropriate and sensitive model to evaluate the effectiveness of preservation strategies and coating performance. In addition, turkey meat was chosen as an alternative to burrata, which is typically packaged in preserving liquid. The presence of governing liquids could enhance the coating performance and make the evaluation more representative of dry-surface applications.

Small cheese samples (50 g) were kindly provided by the dairy farm Parrotta (Putignano, BA, Italy), whereas the meat was purchased from a local market in Foggia (FG, Italy). For each product, two separate tests were carried out for the PET-ZnO and PET-ZnO/Cu films for a total of four shelf life tests. For burrata packaging, four pieces of active film (3 × 3 cm^2^) were added to the brine (75 mL) of each plastic package containing one cheese sample. All packages were closed with a plastic top and then stored at 4 °C for 3 weeks to simulate typical cold chain storage used in retail and domestic refrigeration. A total of 60 burrata samples were prepared to test the PET-ZnO, PET, and control (CNT, no polymer film) considering 10 sampling times with duplicate analyses at each sampling time. Duplicates represent independent experimental repeats. Similarly, PET-ZnO/Cu was tested using an additional 60 burrata samples prepared as described above.

In order to test the films on turkey fillet, two 3 × 3 cm^2^ samples were used to completely cover a meat portion of similar dimension on both sides. The meat was then packaged in appropriately sealed 90 µm high-barrier bags with a pad and stored under refrigerated conditions (4° C) for 2 weeks. Polymer film without any active compound (PET) and fillet without any films (CNT) were also packaged and stored in the same conditions for comparison. A total of 120 fillet samples were prepared for testing both the active coatings and control samples, evaluating 10 samples in duplicate. Duplicates represent independent experimental repeats.

### 2.4. Microbiological Analyses and pH Determination

During storage, the burrata and meat samples were analyzed for microbial quality by the traditional plate count technique. To this aim, approximately 10 g of burrata was diluted with a NaCl solution (9 g/L) and homogenized using a stomacher. Subsequently, serial dilutions were performed in Petri dishes. For total mesophilic bacteria, Peptone from gelatin pancreatic digest was used as the culture medium, incubated at 37 °C for 48 h; for Coliforms, Violet Red Bile Agar was used, incubated at 37 °C for 24 h; *Pseudomonas* spp. were counted on Pseudomonas Agar Base (PAB, Oxoid, Basingstoke, UK) culture medium with the addition of the selective cetrimide fucidin cephaloridine (CFC) supplement, incubated at 25 °C for 48 h; Baird-Parker Agar, supplemented with egg tellurite emulsion, incubated at 37 °C for 48 h, was used to evaluate the growth of *Staphylococcus* spp.; lactococci and lactic acid bacteria were counted on M17 Agar and de Man Rogosa Sharpe Agar (MRS, Oxoid, Basingstoke, UK), respectively, supplemented with cycloheximide (0.1 g/L Sigma, St. Louis, MO, USA), both incubated at 37 °C for 48 h. All microbiological analyses on burrata were performed in duplicate.

For microbiological analysis on meat, approximately 10 g of sample was diluted with a Peptone Water solution (15 g/L) and homogenized using a stomacher. Subsequently, serial dilutions were performed in Petri dishes. For total mesophilic bacteria, Plate Count Agar (PCA, Oxoid) was used as the culture medium, incubated at 30 °C for 48 h; for Enterobacteria, Violet Red Bile Glucose Agar was used, incubated at 37 °C for 24 h; de Man Rogosa Sharpe Agar (MRS, Oxoid), supplemented with cycloheximide (0.1 g/L Sigma) and incubated at 37 °C, was used to count lactic acid bacteria; *Pseudomonas* spp. were counted on Pseudomonas Agar Base (PAB, Oxoid) culture medium supplemented with the selective cetrimide fucidin cephaloridine (CFC) supplement, incubated at 25 °C for 48 h. Baird-Parker Agar, supplemented with egg tellurite emulsion, incubated at 37 °C for 48 h was used to evaluate the growth of *Staphylococcus* spp. All microbiological analyses on meat were conducted in duplicate.

The fitting of experimental data was performed as described in previous research dealing with burrata cheese and meat to calculate the microbiological acceptability limit (MAL) of each sample, to be intended as the time (day) to reach specific microbiological thresholds. The limits were set to 1 × 10^7^ cfu/g for mesophilic bacteria, 1 × 10^6^ cfu/g for *Pseudomonas* spp. and 1 × 10^5^ cfu/g for Coliforms for burrata [22], 1 × 10^7^ cfu/g for lactic acid bacteria, mesophilic bacteria, and *Pseudomanas* spp., 1 × 10^6^ cfu/g for Enterobacteria, and 1 × 10^4^ cfu/g for *Staphylococcus* spp. for meat [24].

The first homogenized dilution of each burrata and meat sample was used to measure the pH (Crison, Barcelona, Spain). Two different samples were used for each measurement.

### 2.5. Sensory Analyses

The samples were submitted to a panel of 7 trained judges to make a quantitative descriptive analysis. The panelists were experts in the sensory evaluation of fresh food before the current study, but were re-trained in the sensory vocabulary and identification of specific attributes in two subsequent sessions (one session/day). For burrata samples, they were asked to evaluate specific sensory parameters such as color, odor, texture, and viscosity as well as the *overall quality* of the entire samples. For the evaluation, a 7-point scale was used, with a score of 4 indicating the limit for product acceptability (1 = lowest score; 7 = highest score) [22]. For the sensory analysis, the samples were presented simultaneously, without using the brine.

The same group of panelists also evaluated the sensory quality of the turkey meat samples. In this case, the judges were asked to give a response about the color, odor, appearance and texture as well as *overall quality*. A 9-point scale was used for the assessment of each specific sensory attribute (1 = lowest score; 9 = highest score; 5 = threshold for acceptability) [25]. According to other studies, sensory quality data were fitted to calculate the Sensory Acceptability Limit (SAL), which represents the time (day) necessary to reach the sensory threshold (score = 4 for burrata samples and score = 5 for meat samples) [22,24].

Sensory evaluations were conducted in a controlled environment at approximately 22 ± 2 °C under neutral, uniform lighting to avoid any visual bias and ensure optimal assessment conditions. For each test, the samples were presented randomly to each panelist with a 3-digit code. A protocol was adopted for protecting the rights and privacy of each panelist during each test. The protocol considered the verbal consent of the participants, without any coercion, and the ability to withdraw from the study at any time. The panelists were fully aware of the study requirements and risks, and no data were released without their knowledge.

### 2.6. Shelf Life Calculation

As reported by other works in the literature, the shelf life of both cheese and meat was calculated considering both microbiological (MAL) and sensory (SAL) acceptability limits [22,24]. The smallest value among the calculated MAL and SAL parameters can be used to define the product shelf life, to be intended as the number of days within which the product remained acceptable.

### 2.7. Statistical Analysis

Data were compared by using the one-way analysis of variance (ANOVA). HSD of Tukey, with the option of homogeneous groups (*p* < 0.05), was carried out to determine significant differences among samples. JMP 18 for Windows (JMP Statistical Discovery LLC 920 SAS Campus Drive, Cary, NC 27513, USA) was used.

## 3. Results and Discussion

### 3.1. Chemical and Morphological Characterization of the Coatings

The SE analysis conducted on Si slices indicates that the thickness of both ZnO and ZnO/Cu coatings was consistent with the theorical values obtained from the ALD growth-per-cycle (GPC, 0.22 nm/cycle) and the Cu sputter deposition rate (2 nm/min). Indeed, the thickness of the ZnO layer was 15.7 ± 0.8 nm, while the thickness of the Cu layer (better Cu/CuO as used in the SE model) was 1.6 ± 0.6 nm, which accounted for a total estimated thickness of 18.3 ± 1.4 nm in the case of the ZnO/Cu samples. The elemental amount of Zn and Cu in the coatings was determined by ICP-MS, and the results are reported in Table 1, together with those obtained from bare Si. Notably, the Zn content in the ZnO and ZnO/Cu coatings was comparable, in agreement with the equal number of ALD cycles employed for ZnO deposition. As for Cu, its amount in the ZnO/Cu sample was 10 times lower than Zn, while it was negligible in the Cu-free samples.

**Figure 1:**
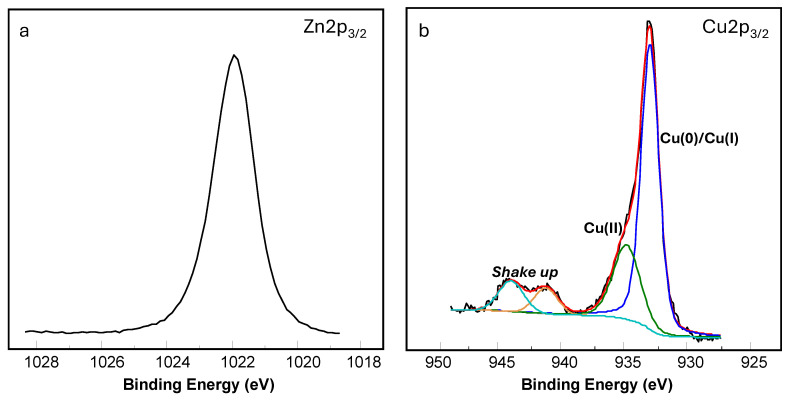
XPS high-resolution spectra of (**a**) Zn2p_3/2_ and (**b**) Cu2p_3/2_ for the ZnO/Cu layer.

In order to gain further insight into the surface chemical composition of the two samples, PET-ZnO and PET-ZnO/Cu, XPS analyses were performed. Figure 1a shows a representative high-resolution spectrum of the Zn2p_3/2_ peak. In both cases, the Zn2p_3/2_ peak was located at 1022.0 eV, which is characteristic of ZnO. Moreover, the modified Auger parameter, calculated from the peak positions of Zn2p_3/2_ and ZnLMM, was 2010.0 eV, further confirming the presence of ZnO. Regarding the Cu content in the ZnO/Cu sample, a Cu/Zn atomic ratio of 0.52 was found. Furthermore, the high-resolution Cu2p_3/2_ spectrum, as shown in Figure 1b, upon peak fitting, exhibited a main component at 932.9 eV, which can be attributed to either Cu(I) or Cu(0) species, and a second component at 934.6 eV, characteristic of CuO. In addition, shake up peaks observed in the 940.0–945.0 eV range confirmed the presence of Cu(II) species. To resolve the ambiguity between Cu(I) and Cu(0), the modified Auger parameter was calculated using the peak positions of Cu2p_3/2_ (932.9 eV) and CuLMM (571.2 eV). A value of 1848.3 eV was obtained, indicating that copper was predominantly present as Cu_2_O, although the presence of metallic copper cannot be completely ruled out. Finally, considering the peak-fitting analysis of Cu2p_3/2_, illustrated in Figure 1b, it can be estimated that Cu(II) accounted for approximately 27% of the total copper content.

**Figure 2:**
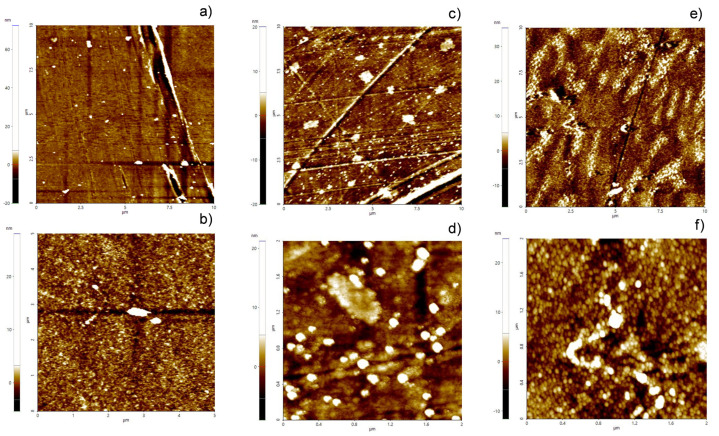
AFM images of (**a**,**b**) bare PET; (**c**,**d**) ZnO deposited on PET; (**e**,**f**) ZnO/Cu deposited on PET.

Additional information regarding the morphology of the deposited films was obtained via AFM analyses of the coated PET substrates. In Figure 2, the AFM images of bare and coated PET are reported. Bare PET presented scratches typical of the fabrication process along with some particulate residues, still visible despite the cleaning procedure. ZnO thin film (Figure 2c) showed some globular aggregates on the surface, presumably due to ZnO deposition. On the other hand, the ZnO/Cu samples (Figure 2e) were characterized by a clearly nanostructured surface, with a higher density of protrusions and brighter features. From Figure 2d,f, which report images taken at a higher magnification of the ZnO and ZnO/Cu samples, respectively, it is possible to gain an insight into the morphology of the two samples. In particular, in the case of the ZnO thin films, some nano-globules with limited aggregation were visible, while the image for ZnO/Cu displayed more rounded features, probably ascribable to the presence of Cu domains, resulting in an overall more textured morphology.

Finally, the leaching of the films in water was investigated by ICP-MS measurements, and the results are summarized in Table 2.

In the control samples (DDW alone and bare PET in DDW), neither Zn nor Cu were detected. When ZnO coated PET (3 cm × 3 cm) was dipped in 50 mL of DDW, 0.103 µg of Zn was released after 4 days and 0.144 µg after 21 days. It is reasonable to argue that most of the release of Zn takes place within 4 days. In the case of the ZnO/Cu samples, comparable amounts of Cu were retrieved after 4 and 21 days in the solution (0.033 µg and 0.04 µg, respectively), confirming the trend highlighted for Zn. With regard to Zn, however, the content in solution after 4 and 21 days for the ZnO/Cu samples was seven times higher than that found for the ZnO coatings. The reason for this behavior is not straightforward; however, it may be attributed to the presence of copper, although further studies are needed to confirm this hypothesis.

It should be noted that the leaching experiments reported here were performed in water as a simplified extraction medium. In real food matrices such as cheese brine or meat, the presence of proteins, lipids, and salts may alter metal ion speciation and potentially affect dissolution kinetics. However, since ZnO and Cu were deposited as conformal ALD thin films firmly anchored to the PET surface, nanoparticle detachment is unlikely, and metal release is expected to occur mainly via slow ionic dissolution. Future studies will investigate migration behavior using standardized food simulants to better approximate real food systems.

### 3.2. Shelf Life Tests on Burrata Cheese

**Figure 3:**
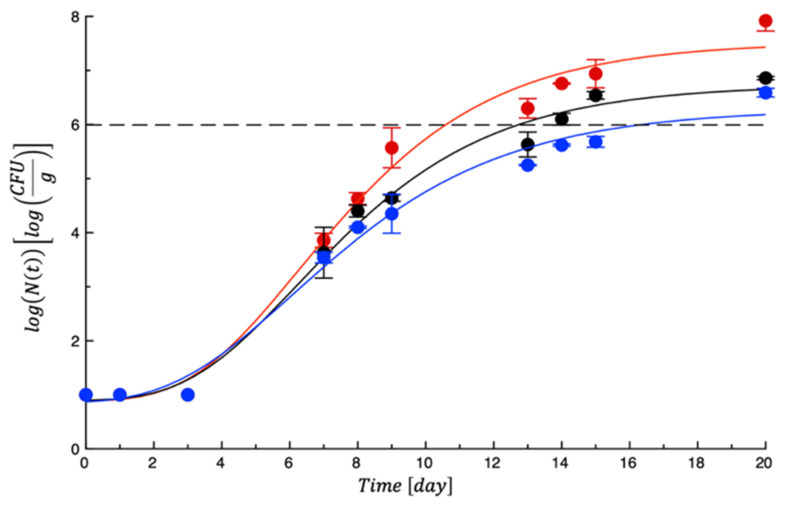
Evolution of *Pseudomonas* spp. in burrata samples during storage. Data are the means ± standard deviations. The curves are the best fit to the experimental data. Dashed line represents the microbial threshold. Red = CTN; Black = PET; Blue = PET-ZnO.

For testing the efficacy of the PET-ZnO film on cheese, the samples were packaged with and without films and monitored for about 3 weeks. During the refrigerated storage, different microbial and fungal growths were recorded. Figure 3 shows the *Pseudomonas* spp. proliferation in burrata samples during storage. As can be seen, the microorganisms proliferated in all cheeses and reached the maximum level of 1 × 10^6^ cfu/g after the first week of storage in the control sample (CNT) and in the sample with the control film (PET), whereas it was after 2 weeks in the sample with active film (PET-ZnO). This evidence demonstrates that the presence of ZnO prevented microbial proliferation. The effect is not surprising because other literature findings have reported that ZnO can inhibit alterative and pathogenic bacteria. Qu et al. [26] studied a green, one-step approach for the in situ synthesis of ZnO particles within a sodium caseinate and carboxymethyl cellulose matrix, with functionality enhanced by thymol essential oil. When applied to cheddar cheese, the film significantly suppressed microbial growth and effectively prevented external contamination, maintaining product safety over 20 days under refrigerated storage.

The primary mechanism of action involves the generation of ROS, which compromise bacterial cell membranes, inhibit protein synthesis, and disrupt DNA replication through oxidative stress [27]. Due to the electrostatic forces, ZnO nanoparticles are strongly attracted to the bacterial cell membrane, and thanks to UV and visible light activation, induce the formation of the potent antimicrobial agent H_2_O_2_ [28]. Furthermore, their antibacterial activity is exerted by releasing Zn^2+^ ions, which cause the coagulation of bacterial proteins and the inactivation of bacterial enzymes [29]. In the current study, to provide quantitative information about the film efficacy, the fitting of experimental data allowed us to calculate the MAL value for each sample, which indicates that the number of days within burrata remained acceptable. In Table 3, the MAL related to *Pseudomonas* spp. is reported for the three investigated samples. As can be inferred, both CNT and PET became unacceptable after 10 and 12 days, respectively, whereas the cheese packaged with the active film remained acceptable for less more than 15 days.

Mesophilic bacteria and Coliforms have specific microbial thresholds in pasta filata cheese such as burrata, but these limits were never reached during the entire monitoring period, regardless of the packaging conditions adopted. Data are reported in the Supplementary Materials (Figure S1). For this reason, data were not elaborated by the fitting procedure, and MAL values higher than the monitoring period (20 days) are listed in Table 3. Coliforms are usually considered by food manufacturers as hygiene indicators of the sanitary quality of cheese because their growth is mainly due to the use of contaminated raw milk, lack of pasteurization, use of poorly controlled fermentation as well as insufficient storage time and maturation conditions [30]. Beigmohammadi et al. [31] tested the antibacterial potential of low-density polyethylene (LDPE) films incorporated with ZnO nanoparticles on the growth of Coliform bacteria in ultra-filtrated cheese and found that the number of surviving bacteria decreased after 4 weeks of storage at 4 ± 0.5 °C.

**Figure 4:**
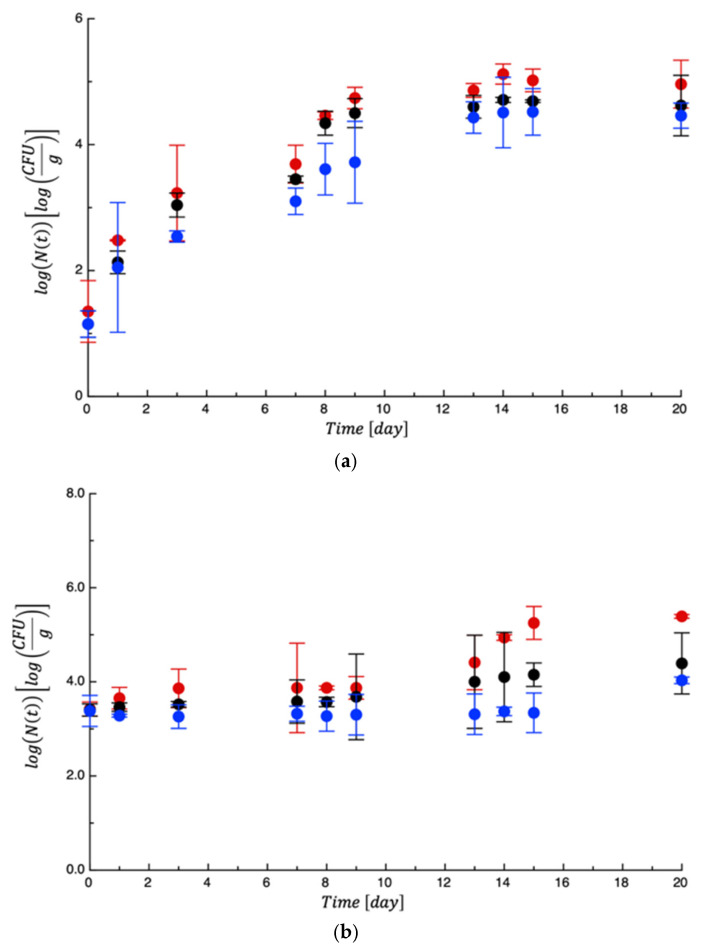
Evolution of lactic acid bacteria (**a**) and *Lactococcus* (**b**) in burrata samples during storage. Data are the means ± standard deviations. Red = CTN; Black = PET; Blue = PET-ZnO.

*Staphylococcus* spp. and yeasts grew in all samples until reaching about 10^5^ cfu/g and 10^6^ cfu/g, respectively, (Figure S2), but the increase in these microorganisms did not seem to be strongly influenced by the antimicrobial properties of the active films adopted in the packaging. The evolution of lactic acid bacteria and *Lactococcus* bacteria are reported in Figure 4a,b, where it is possible to see that all the samples presented a very similar trend, and therefore the proliferation did not strongly depend on the packaging. The advantage of ZnO can be added to its other positive aspects such as stability and broad-spectrum antimicrobial activity against spoilage microorganisms [32].

**Figure 5:**
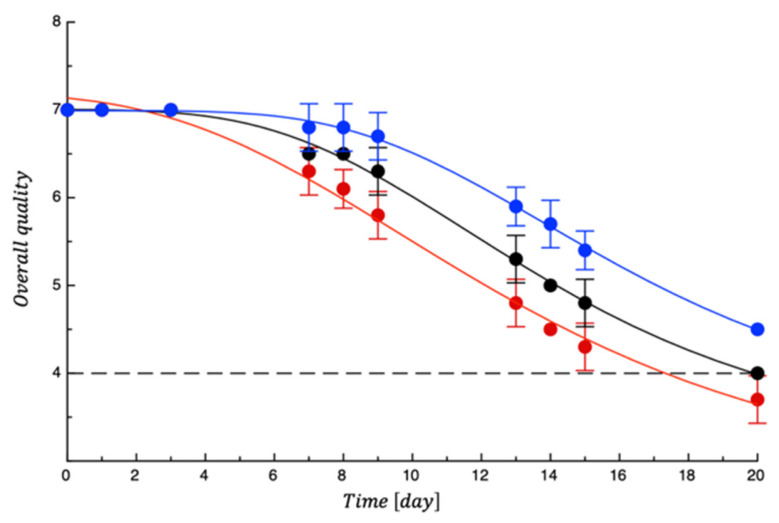
Evolution of burrata samples’ overall quality during storage. Data are the means ± standard deviations. The curves are the best fit to the experimental data. Dashed line represents the sensory threshold. Red = CTN; Black = PET; Blue = PET-ZnO.

The pH remained around 7 in all samples and for the entire monitoring period (*p* > 0.05). Since the pH is directly related to the chemical changes associated with the protein network of cheese curd, a lack of changes in pH is expected to prevent an increase in texture hardness [33]. As a fact, Figure 5 reports the burrata cheese sensory acceptability. The graph highlights that cheese remained acceptable for more than 2 weeks at least. As expected, the sensory scores decreased with an increase in the storage time, but the degree of quality change of cheese was dependent on the type of packaging films. Significant differences among samples were recorded (*p* < 0.05). The control sample became unacceptable slightly earlier than the burrata packaged with PET, but the most important difference (*p* < 0.05) was with the samples packaged with active film, which became unacceptable well after 3 weeks of storage. Generally, based on the obtained results, the packaging of burrata with an active nanocomposite film protected the sensory parameters of the samples during storage time due to the control of microbial spoilage. This effect is not new in the literature because other studies have also found similar results using a nanocomposite film based on ZnO [30,34].

Table 3 also reports the data of SAL calculated from fitting to the experimental data. As can be seen, the acceptability increased up to 24 days in the sample with active film, thus demonstrating an improvement in sensory quality when the microbial proliferation was delayed.

Comparing the data of MAL and SAL in Table 3, it is possible to infer the shelf life as the lowest value among them. As can be seen, shelf life values depend on *Pseudomonas* spp. proliferation, and therefore CNT and PET became unacceptable after 10 and 12 days, respectively, whereas the active sample remained acceptable for 16 days. In accordance with these results, Youssef et al. [35] reported that the novel chitosan/carboxymethyl cellulose/ZnONPs bio-nanocomposite film improved the shelf life of white soft cheese. Accordingly, Korany et al. [36] assessed that gelatin-based ZnO nanoparticle bio-nanocomposite coatings applied to Talaga cheese promoted a shelf life prolongation.

**Figure 6:**
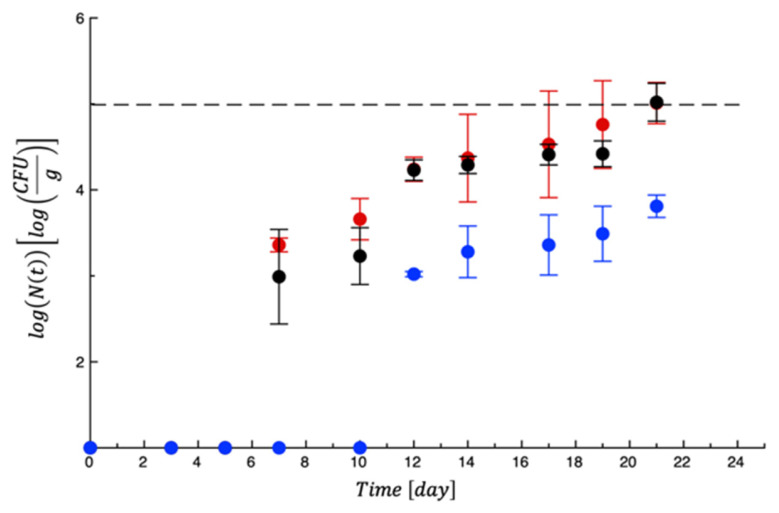
Evolution of Coliforms in burrata samples during storage. Data are the means ± standard deviations. Dashed line represents the microbial threshold. Red = CTN; Black = PET; Blue = PET-ZnO/Cu.

When the new test was carried out with PET-ZnO/Cu, new burrata samples were packaged and monitored again for 3 weeks, also considering a control sample (CNT) without film and a sample with neat PET. As in the previous test, cell counts of mesophilic bacteria did never reach any threshold (Figure S3). Coliforms are reported in Figure 6. In the graph, it is possible to see that for about 2 weeks, the microbial proliferation was very limited. It is also striking to observe that the active film greatly controlled Coliform proliferation.

A similar trend was recorded for *Pseudomonas* spp. (Figure S4), whose counts reached the threshold around 21 days in both the controls and never reached any limit in the sample with active film. This efficacy can be justified by the synergistic effect between ZnO and Cu. Synergy may arise from simultaneous multi-site attacks: ZnO primarily destabilizes membranes and cell walls, Cu ions penetrate cells and damage intracellular biomolecules, and combined ion release may disrupt metabolic pathways more efficiently. Recent studies suggest that nanoparticles can simultaneously target bacteria through various mechanisms [37]. Said Elshahat et al. [38], who recently investigated green-synthesized copper oxide nanoparticles for food packaging applications, suggested three main key approaches to justify the antimicrobial mechanism: the release of Cu^2+^ ions from the nanoparticles, direct interaction with bacterial cells, and the production of ROS. Therefore, the presence of copper can catalyze redox cycling reactions that amplify ROS generation initiated by ZnO. This dual ROS pathway may overwhelm microbial antioxidant defense systems, accelerate lipid peroxidation in cell membranes, and increase intracellular oxidative damage. Longano et al. [39] synthesized metal nanoparticles based on Cu by means of laser ablation, as a rising and easy route to prepare nanostructures without any capping agent in a liquid environment. The nanocomposite of copper nanoparticles embedded in polylactic acid was found to be effective against strains of *Pseudomonas* spp. In another study, these nanocomposite coatings embedding copper nanoparticles were found to be effective in preventing microbial growth in fiordilatte cheese [40]. The results were not obvious because when chemically complex foods were selected, antimicrobial polymers with metal nanoparticles were found mostly ineffective in reducing bacterial populations, probably due to the inactivation of metals by proteins [41]. Gvozdenko et al. [42] investigated the possibility of using methylcellulose films modified with CuO nanoparticles for the packaging of hard cheese. These authors found that the active films inhibited the growth of fungi and bacteria in experimental cheese samples because during storage, CuO nanoparticles migrated to the product from the film.

In the current study, the calculated MAL values were around 21 days for the two control samples and higher than 21 days with the active packaging.

*Staphylococcus* spp. and yeasts recorded very similar trends to the previous test, without important differences among samples (*p* > 0.05) (Figure S5). With regards to lactic acid bacteria, the proliferation did not suffer the influence of ZnO and Cu in the packaging system, thus promoting the potential of this solution, which exerts antimicrobial effects against spoilage microorganisms without affecting the typical dairy flora. The pH of the samples remained between 6 and 7 in all cheeses and for the entire monitoring period (*p* > 0.05).

**Figure 7:**
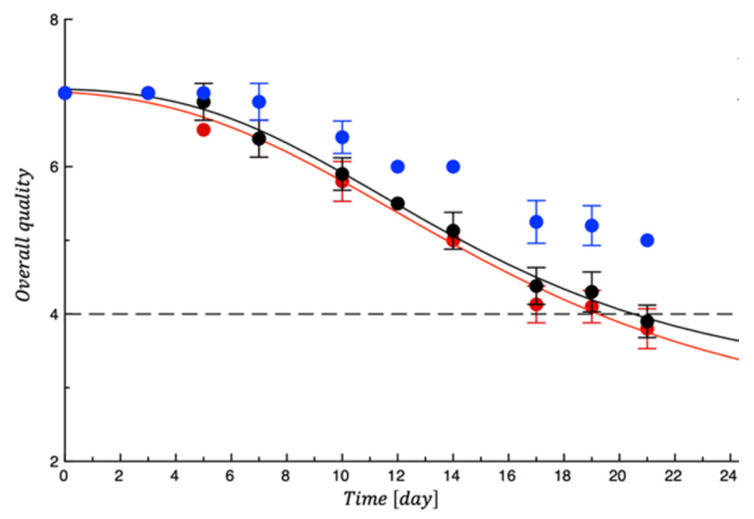
Evolution of burrata samples’ overall quality during storage. Data are the means ± standard deviations. The curves are the best fit to the experimental data. Dashed line represents the sensory threshold. Red = CTN; Black = PET; Blue = PET-ZnO/Cu.

The sensory evolution is reported in Figure 7. In the graph, it is possible to observe that while the control samples became unacceptable within 3 weeks, burrata with PET-ZnO/Cu also still maintained a good quality at the last sampling time. As reported in the previous test, the inhibition of microbial proliferation also allowed for better sensory properties to be maintained [43]. Considering that from the microbiological point of view no limits were reached for burrata cheese, the shelf life in this second test was limited by the sole SAL values. Therefore, both controls recorded a shelf life around 20 days, whereas the active film PET-ZnO/Cu maintained samples acceptable for more than 21 days.

## 4. Shelf Life Tests on Turkey Fillet

**Figure 8:**
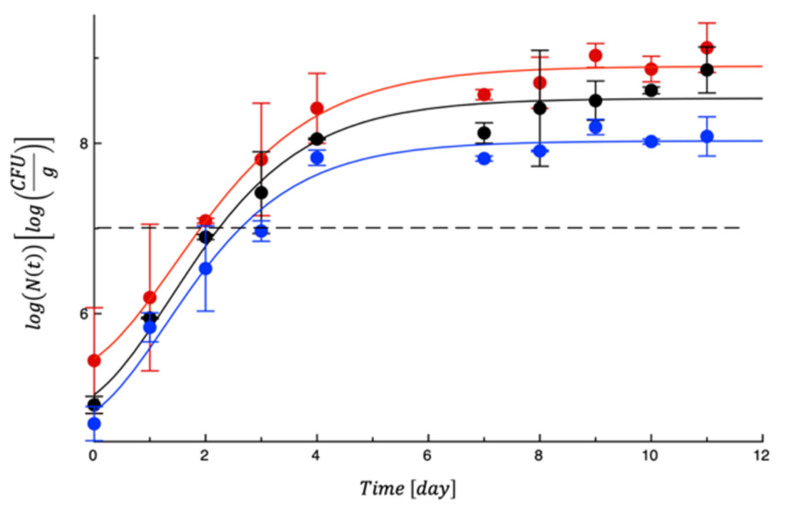
Evolution of *Pseudomonas* spp. in turkey meat samples during storage. Data are the means ± standard deviations. The curves are the best fit to the experimental data. Dashed line represents the microbial threshold. Red = CTN; Black = PET; Blue = PET-ZnO.

To test the efficacy of the PET-ZnO film on turkey meat, active and control samples were monitored for 11 days. During this period, different microbial growth rates were observed. As can be seen in Figure 8, in the control meat and in the sample with the control film (PET), *Pseudomonas* spp. grew very rapidly and reached the maximum concentration after about two days from the start of the test, while in the sample with the PET-ZnO, the microbial proliferation was slowed down. Amjadi et al. [30] prepared gelatin (G)-based nanocomposite films containing different concentrations of cellulose nanofiber (CNF) and ZnO-NPs at 1%, 3%, 5%, and 7% (*w*/*w* of gelatin) by the casting method. Inoculated on chicken fillets, G/5% CNF/5% ZnO showed a remarkable antimicrobial efficacy against *Pseudomonas fluorescens*. Further applications of ZnO nanoparticles as meat packaging materials can be found in the recent review of Smaoiu et al. [43], which discussed ZnO-NP synthesis, antibacterial potential, and functional application as meat packaging. These authors explored the physicochemical properties of ZnO-NPs synthesized through different routes with a special focus on the antibacterial mechanisms that underlie synthesis parameters, thus supporting the evidence that ZnO-NPs enhance stored meat product quality by microflora growth limitation and lipid/protein oxidation delay.

In Table 4, the MAL values related to *Pseudomonas* spp. for the 3 tested films are presented.

**Figure 9:**
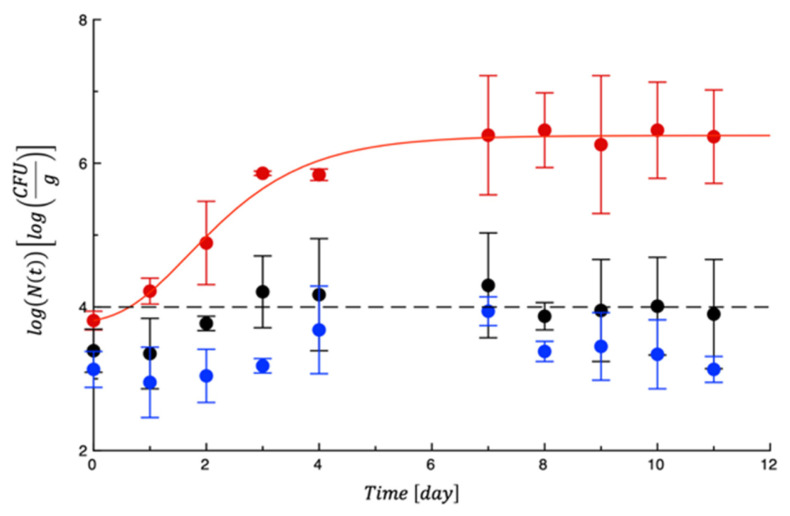
Evolution of *Staphylococcus* spp. in turkey meat samples during storage. Data are the means ± standard deviations. The curves are the best fit to the experimental data. Dashed line represents the microbial threshold. Red = CTN; Black = PET; Blue = PET-ZnO.

For mesophilic bacteria, lactic acid bacteria, and Enterobacteria, the thresholds were never reached by either the control or the active samples (Figure S6), even though a certain effect of ZnO on these microorganisms was observed, also supported by other findings in the literature recorded in poultry meat [44,45]. For these microbial counts, the MAL values were indicated higher than 11 days (Table 4). For *Staphylococcus* spp., as reported in Figure 9, a significant difference could be observed between the control sample and meat packaged in the presence of the film. In fact, as can be seen, the sample without any film exceeded the microbiological acceptability limit (1 × 10^4^ cfu/g) at the beginning of the test, while in the samples covered with PET or PET-ZnO, the proliferation was abundantly delayed and remained around 10^4^ cfu/g for the entire period. Table 4 lists the MAL values.

The pH remained between 6 and 7 in all samples and for the entire monitoring period (*p* > 0.05). At the beginning of storage, the pH slightly declined due to the creation of lactic acid and other acidic compounds. As storage progressed, the pH increased due to protein decomposition by endogenous enzymes and microorganisms [46]. Throughout storage, turkey fillet packaged with the ET-ZnO film exhibited a significantly smaller pH increase than the controls (*p* < 0.05). Among the experimental groups, the active film resulted in the least pH variation, indicating that ZnO effectively inhibited microbial growth and subsequently delayed the rise in pH.

**Figure 10:**
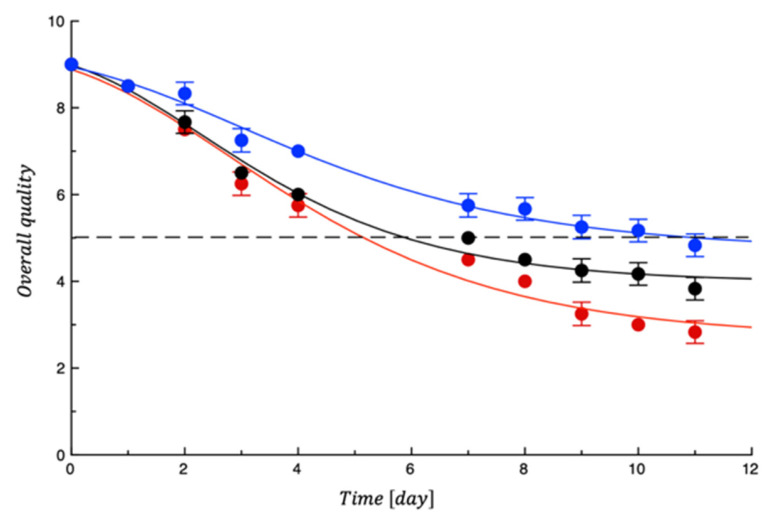
Evolution of turkey meat samples’ overall quality during storage. Data are the means ± standard deviations. The curves are the best fit to the experimental data. Dashed line represents the sensory threshold. Red = CTN; Black = PET; Blue = PET-ZnO.

The graph in Figure 10 shows the overall quality trend of the tested turkey fillet samples. As we can see, the scores of sensory attributes in all the evaluated samples showed a decreasing trend. However, the rate of sensory loss was slower for the active coated samples compared to the control meat. A progressive off-color, off-odor, and texture loss was started in the samples because of spoilage development but the presence of ZnO allowed the meat to be preserved for longer. There are many studies in agreement with our results about the effect of active films on the preservation of food in terms of sensory properties. These findings are related to the progress of microbial growth in the control samples during storage, which are involved in organoleptic losses in comparison to samples coated with the active film [47]. Table 4 also reports the data of SAL calculated from fitting to the experimental sensory data. As can be seen, the acceptability increased up to 11 days in the sample with active film, thus demonstrating an improvement in sensory quality.

As reported in Table 4, the shelf life values of turkey meat depend on the proliferation of *Staphylococcus* spp. and *Pseudomonas* spp.; in fact, data show that the control remained acceptable for less than 1 day for undesired *Staphylococcus* spp. proliferation, whereas the samples with film remained acceptable for more than 2 days for the high growth of *Pseudomonas* spp. The difference between the control sample and active film was significant (*p* < 0.05), but similar effects were found between the two meat samples covered with the neat PET and with the active film.

Further research will be necessary for a deep investigation of this result, but the scientific literature suggests some explanations. One of the factors that influenced the bactericidal action of the films is the food matrix. The interaction of the different components, both nutritious and non-nutritive, generally gives the meat specific physicochemical characteristics that could create a suitable or hostile environment for the meat against the action of an antibacterial agent [48].

**Figure 11:**
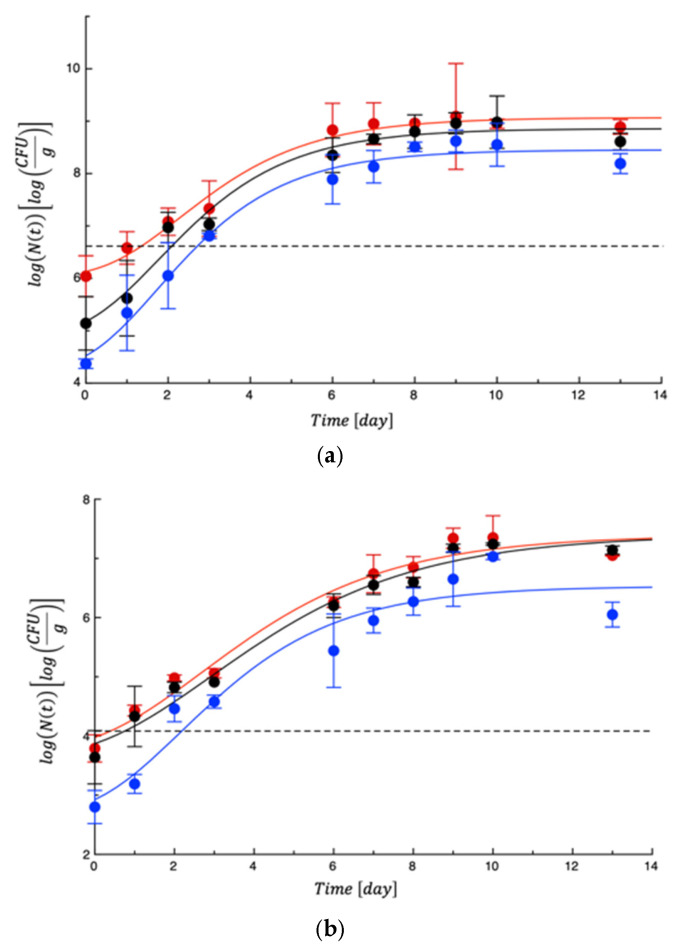
Evolution of *Pseudomonas* spp. (**a**) and *Staphylococcus* spp. (**b**) in turkey meat samples during storage. Data are the means ± standard deviations. The curves are the best fit to the experimental data. Dashed line represents the microbial threshold. Red = CTN; Black = PET; Blue = PET-ZnO/Cu.

To test the PET-ZnO/Cu films, new meat samples were prepared, packaged, and monitored for approximately 13 days. Again, mesophilic bacteria and lactic acid bacteria showed moderate proliferation and never reached any limit (Figure S7). A very similar evolution was observed for Enterobacteria (Figure S7). On the other hand, *Pseudomonas* spp. and *Staphylococcus* spp. grew rapidly, as in the previous test. As can be seen in Figure 11, while the two control samples became unacceptable within the first 2 days of storage, the active film was able to delay microbial growth. Table 5 reports the MAL values. Compared to the previous test, the effects of the active film were more marked due to the synergistic effects between ZnO and Cu derived from amplified ROS generation, dual metal ion toxicity, multi-target cellular damage, enhanced membrane interaction, and broader antimicrobial coverage [49]. As a fact, ZnO nanoparticles show strong activity against Gram-positive bacteria, while copper exhibits significant effects against Gram-negative bacteria and fungi. Their combination may expand the antimicrobial spectrum and improve efficacy against mixed microbial communities typical of food spoilage, as assessed in other literature works [50]. The concurrent damage to membrane integrity, DNA, proteins, and enzymatic systems reduces the probability of microbial adaptation or resistance development.

The pH of the samples remained around 6 in all samples for the entire monitoring period (*p* > 0.05).

**Figure 12:**
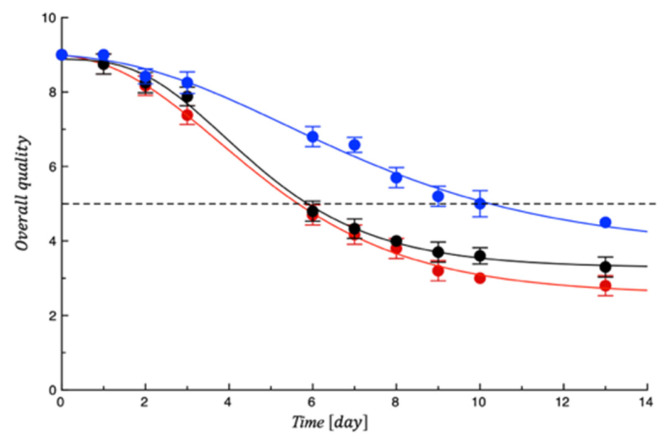
Evolution of turkey meat samples overall quality during storage. Data are means ± standard deviations. The curves are the best fit to the experimental data. Dashed line represents the sensory threshold. Red = CTN; Black = PET; Blue = PET-ZnO/Cu.

Figure 12 shows the trend of the sensory quality of the tested meat fillets. It is possible to observe a very similar trend for both the control samples that became unacceptable after 6 days of storage compared to meat samples covered with PET-ZnO/Cu, which instead remained acceptable until the 10th day of storage. The scores obtained for the active sample were significantly higher (*p* < 0.05) than both the controls, indicating the functional film’s efficiency in preventing quality decay, above all controlling meat appearance and off-odor that are the main factors responsible for meat unacceptability [50]. This result is in good agreement with the microbiological test results. Table 5 also reports the SAL values calculated by the fitting procedure.

**Figure 13:**
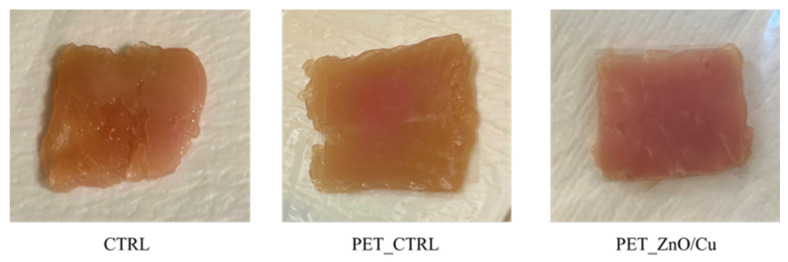
Turkey meat samples stored for 10 days to 4 °C.

Figure 13 shows the images of the samples after 10 days of storage. In the photo, the reader can see that the sample with the active film maintained the typical pink color of meat compared to the control samples that appeared slightly yellow. These results show that the active film, by forming a layer on the surface of the meat, may be able to reduce the pigmentation rate and maintain the original color.

Comparing the data reported in Table 5, it is possible to infer that microbial proliferation, and in particular *Staphylococcus* spp. Growth, provoked the end of the shelf life. Table 5 also highlights that the active film was effective in slowing down bacterial growth and consequently preserving the sensory quality of meat over time. Thanks to the inhibitory effect on the meat spoilage, a shelf life extension was recorded. Specifically, the shelf life of the fillet with active film was more than double with respect to the control samples. Similar findings were reported in the literature on pork meat. Roy et al. [51] developed copper-modified zinc oxide nanoparticles added to gelatin/agar-based multifunctional films, loaded with a clove essential oil Pickering emulsion, and tested the active film on the shelf life of wrapped pork meat stored at 10 °C, demonstrating a great inhibition of the multifunctional film on microbial counts and a consequent significant shelf life prolongation compared to the unwrapped control sample.

## 5. Conclusions

Active antimicrobial coatings were deposited on PET by means of ALD and plasma sputtering and tested on two different fresh foods, burrata cheese and turkey fillets. The composite films consisted of PET coated with ZnO and ZnO/Cu layers as thin as about 15 and 18 nm, respectively. XPS confirmed the presence of Zn in zinc oxide form and of Cu as copper oxide (Cu_2_O and CuO), while ICP-MS analysis of the digested coating allowed for their quantitation. In particular, the amount of zinc was about 10 µg/cm^2^ on both samples, whereas Cu on the ZnO/Cu sample was around 130 ng/cm^2^. Their effects were tested against microbial proliferation and sensory decay during a proper storage period, under refrigerated conditions. The two films exerted different effects because the presence of Cu further increased the film performance. It is also worth noting that cheese and meat are different foods, with different initial contamination levels and different microbial evolutions. Consequently, different effects of the films were recorded. The PET-ZnO tested on burrata cheese was found to be effective in promoting some days of shelf life prolongation, mainly due to antimicrobial effects against *Pseudomonas* spp. When the PET-ZnO/Cu was tested, the effects were amplified. During the 21 days of monitoring, burrata cheese packaged with active film never reached any threshold, remaining acceptable for more than 3 weeks compared to the control samples, which were found to be acceptable up to 19–20 days. When tests were carried out on turkey fillets, the effects of the two active films were more marked because meat was more contaminated. In both tests with PET-ZnO and PET-ZnO/Cu, the loads of *Staphylococcus* spp. grew rapidly in the control sample, which for this reason became unacceptable within the first day of storage. The presence of the coating on the PET film promoted a significant shelf life prolongation because it inhibited spoilage proliferation and consequently enhanced product sensory quality. Best results were recorded in the last test when Cu was combined with ZnO, and both microbial counts and sensory quality were delayed. Samples remained acceptable for more than 2 days compared to less than 1 day in both control meat samples.

## Figures and Tables

**Table 1:** Zn and Cu amounts by ICP-MS in bare Si, ZnO-coated Si, and ZnO/Cu-coated Si.

	Zn (µg/cm^2^)	Cu (µg/cm^2^)
Si	0.020 ± 0.001	0.003 ± 0.001
ZnO	9.86 ± 0.05	0.003 ± 0.001
ZnO/Cu	10.20 ± 0.05	0.13 ± 0.01

**Table 2:** ICP-MS quantitation of Zn and Cu levels in water after soaking of PET-ZnO and PET-ZnO/Cu.

Soaking Time (Day)	Samples	Mass Released (µg)	
		Cu	Zn
4	PET-ZnO	//	0.103
	PET-ZnO/Cu	0.033	0.73
21	PET-ZnO	//	0.144
	PET-ZnO/Cu	0.04	0.942

**Table 3:** Microbiological and sensory acceptability limit (MAL and SAL) and shelf life value of burrata.

		MAL [Day]		SAL [Day]	Shelf Life [Day]
	Mesophilic Bacteria (1 × 10^7^)	*Pseudomonas* spp. (1 × 10^6^)	Coliforms (1 × 10^5^)	Score > 4	
CNT	>20	10.6 ± 0.5 ^b^	>20	17.1 ± 0.186 ^c^	10.6 ± 0.5 ^b^
PET	>20	12.8 ± 0.6 ^b^	>20	19.9 ± 0.510 ^b^	12.7 ± 0.6 ^b^
PET-ZnO	>20	16.4 ± 2.1 ^a^	>20	24.4 ± 1.133 ^a^	16.4 ± 2.1 ^a^

CNT = burrata without film; PET = burrata with no active film; PET-ZnO = burrata with active film. ^a,b,c^ Data in the same column with different superscript letter are significantly different (*p* < 0.05).

**Table 4:** Microbiological and sensory acceptability limit (MAL and SAL) and shelf life value of turkey meat.

		MAL [Day]				SAL [Day]	Shelf Life [Day]
	Mesoph. (1 × 10^7^)	*Pseud.* (1 × 10^7^)	Enterob. (1 × 10^6^)	LAB (1 × 10^7^)	*Staphyl.* (1 × 10^4^)	Score > 5	
CNT	>11	1.9 ± 0.1 ^b^	>11	>11	0.6 ± 0.2	5.1 ± 0.3 ^b^	0.6 ± 0.2b ^b^
PET	>11	2.2 ± 0.2 ^a,b^	>11	>11	>11	5.9 ± 0.3 ^b^	2.2 ± 0.2 ^a^
PET-ZnO	>11	2.6 ± 0.2 ^a^	>11	>11	>11	11.0 ± 1.4 ^a^	2.6 ± 0.2 ^a^

CNT = turkey meat without film; PET = turkey meat with no active film; PET-ZnO = turkey meat with active film. Mesoph. = mesophilic bacteria; *Pseud.* = *Pseudomonas* spp.; Enterob. = Enterobacteriaceae; LAB = lactic acid bacteria; *Staphyl.* = *Staphylococcus* spp. ^a,b^ Data in the same column with different superscript letter are significantly different (*p* < 0.05).

**Table 5:** Microbiological and sensory acceptability limit (MAL and SAL) and shelf life value of turkey meat.

		MAL [Day]				SAL [Day]	Shelf Life [Day]
	Mesoph. (1 × 10^7^)	*Pseud.* (1 × 10^7^)	Enterob. (1 × 10^6^)	LAB (1 × 10^7^)	*Staphyl.* (1 × 10^4^)	Score > 5	
CNT	>13	2.0 ± 0.2 ^b^	11.5 ± 18	>13	0.1 ± 0.7 ^b^	5.7 ± 0.08 ^b^	0.1 ± 0.7 ^b^
PET	>13	2.6 ± 0.2 ^b^	>13	>13	0.5 ± 0.5 ^b^	5.9 ± 0.1 ^b^	0.5 ± 0.5 ^b^
PET-ZnO/Cu	>13	3.2 ± 0.2 ^a^	>13	>13	2.1 ± 0.4 ^a^	10.2 ± 0.4 ^a^	2.0 ± 0.3 ^a^

CNT = turkey meat without film; PET = turkey meat with no active film; PET-ZnO/Cu = turkey meat with active film. Mesoph. = mesophilic bacteria; *Pseud.* = *Pseudomonas* spp.; Enterob. = Enterobacteriaceae; LAB = lactic acid bacteria; *Staphyl.* = *Staphylococcus* spp. ^a,b^ Data in the same column with different superscript letter are significantly different (*p* < 0.05).

## Data Availability

The original contributions presented in this study are included in the article/Supplementary Materials. Further inquiries can be directed to the corresponding authors.

## Supplementary Materials

The following supporting information can be downloaded at: https://www.mdpi.com/article/10.3390/polym18060751/s1, Figure S1: Evolution of mesophilic bacteria (a) and Coliforms (b) in burrata samples during storage. Figure S2: Evolution of *Staphylococcus* spp. (a) and yeasts (b) in burrata samples during storage. Figure S3: Evolution of mesophilic bacteria in burrata samples during storage. Figure S4: Evolution of *Pseudomonas* spp. in burrata samples during storage. Figure S5: Evolution of *Staphylococcus* spp. (a) and yeasts (b) in burrata samples during storage. Figure S6: Evolution of mesophilic bacteria (a), lactic acid bacteria (b), and Enterobacteria (c) in turkey meat samples during storage. Figure S7: Evolution of mesophilic bacteria (a), lactic acid bacteria (b), and Enterobacteria (c) in turkey meat samples during storage.
