# Supplementary material for: Atomic Layer Deposition of ZnO and ZnO/Cu Coatings for Fresh Food Packaging Application
Source: Polymers (Basel). 2026 Mar 19;18(6):751. doi: 10.3390/polym18060751 (PMC13030190; doi:10.3390/polym18060751)

Supporting Data for the Article

Atomic Layer Deposition of ZnO and ZnO/Cu Coatings for Fresh Food Packaging Application

Adriana Lordi, Regina Del Sole, Fabio Palumbo, Alberto Perrotta, Francesco Fracassi, Marianna Roggio, Antonella Milella, Amalia Conte and Matteo Alessandro Del Nobile

Figure S1: Evolution of mesophilic bacteria (a) and Coliforms (b) in burrata samples during storage. Data are means ± standard deviations. Red = CTN; Black = PET; Blue = ZnO/PET

a)

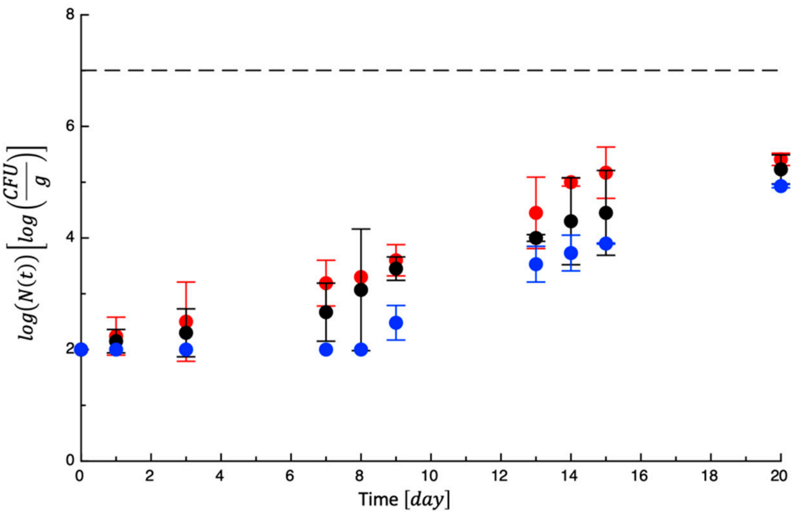

b)

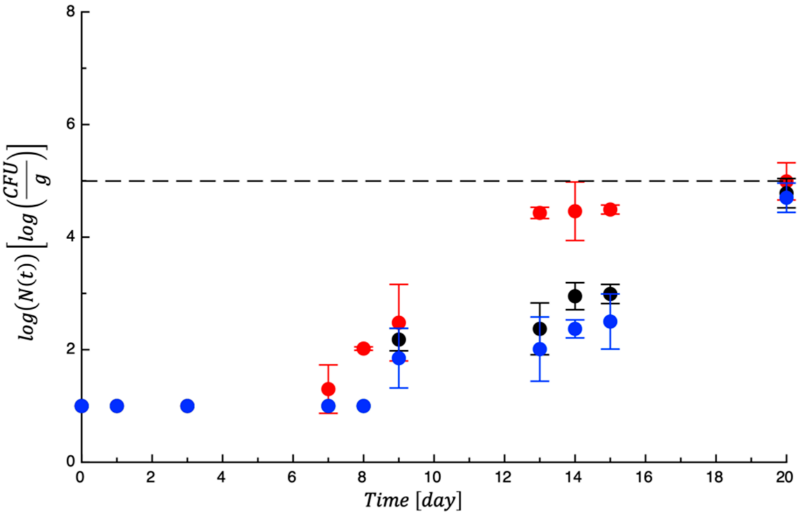

Figure S2: Evolution of *Staphylococcus* spp. (a) and yeasts (b) in burrata samples during storage. Data are means ± standard deviations. Red = CTN; Black = PET; Blu = ZnO/PET

a)

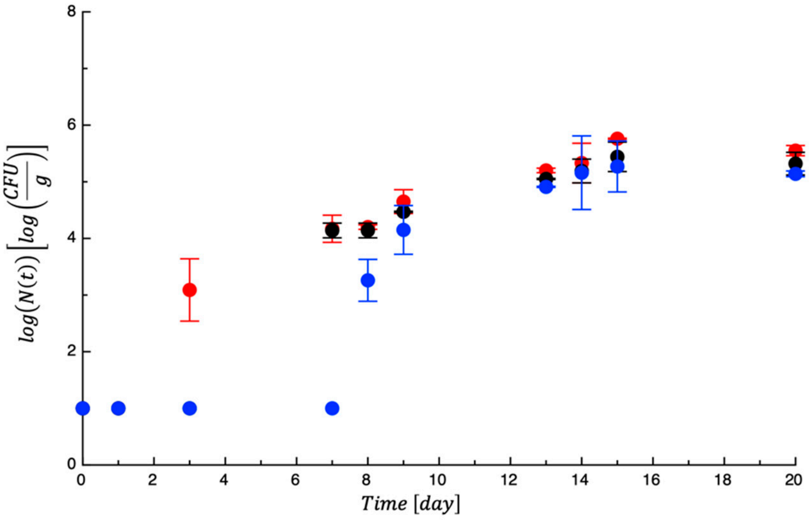

b)

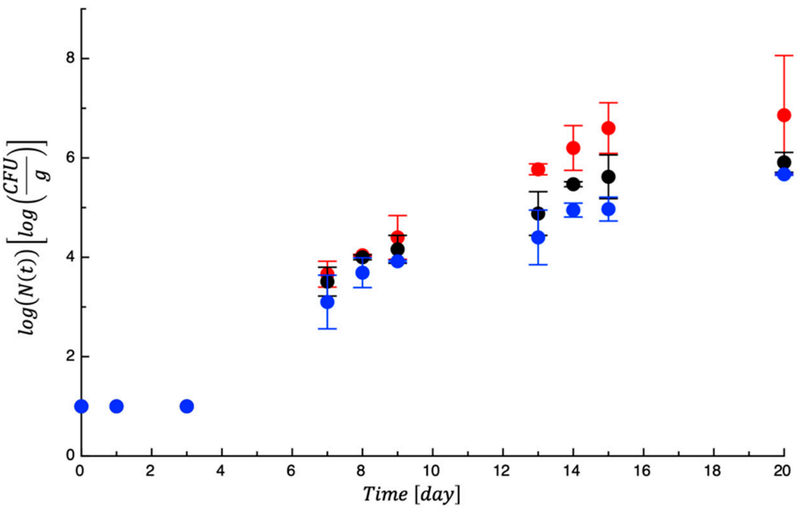

Figure S3: Evolution of mesophilic bacteria in burrata samples during storage. Data are means ± standard deviations. Red = CTN; Black = PET; Blu = PET-ZnO/Cu

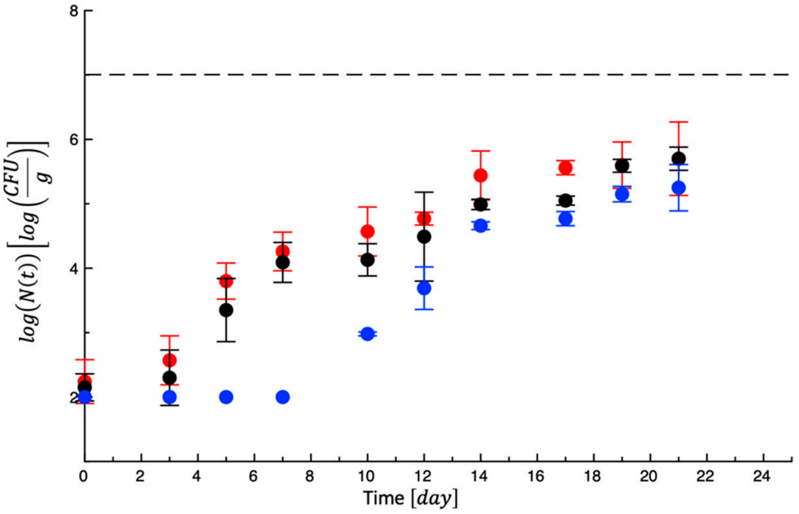

Figure S4: Evolution of *Pseudomonas* spp. in burrata samples during storage. Data are means ± standard deviations. Red = CTN; Black = PET; Blu = PET-ZnO/Cu

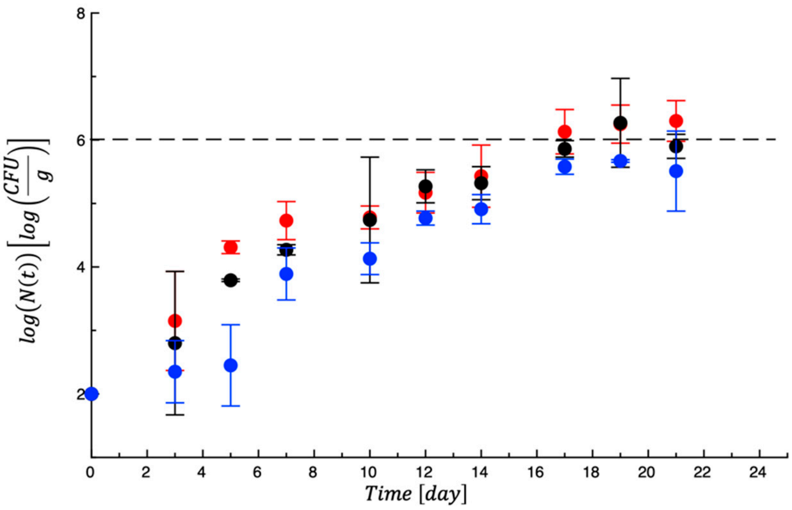

Figure S5: Evolution of *Staphylococcus* spp. (a) and yeasts (b) in burrata samples during storage. Data are means ± standard deviations. Red = CTN; Black = PET; Blu = PET-ZnO/Cu

a)

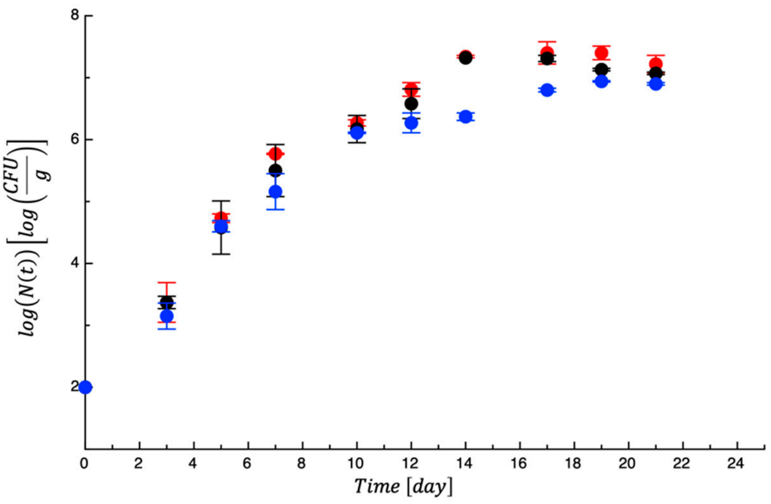

b)

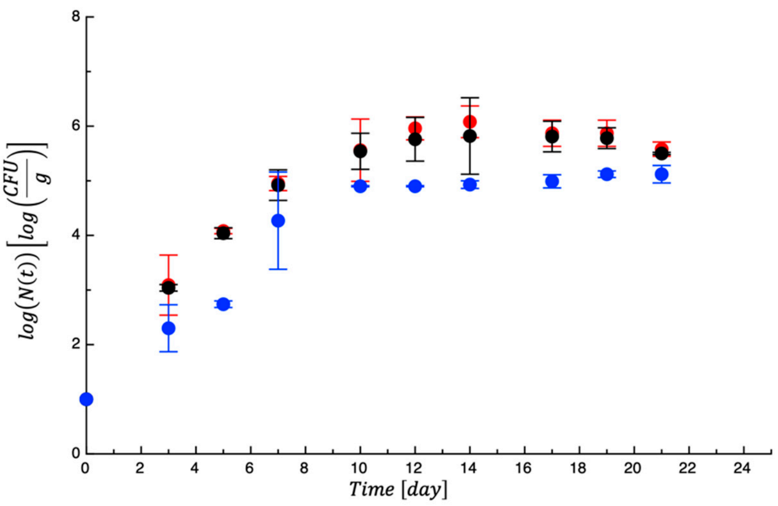

Figure S6: Evolution of mesophilic bacteria (a), lactic acid bacteria (b) and Enterobacteria (c) in turkey meat samples during storage. Data are means ± standard deviations. Red = CTN; Black = PET; Blu = PET-ZnO

a)

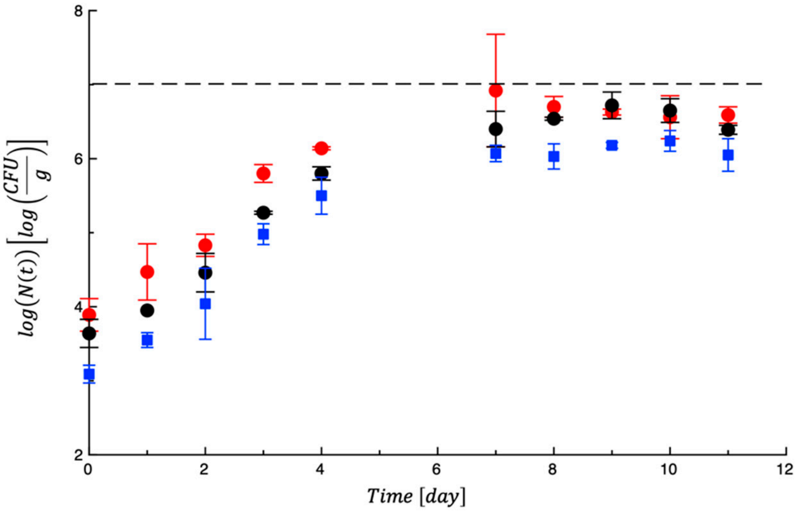

b)

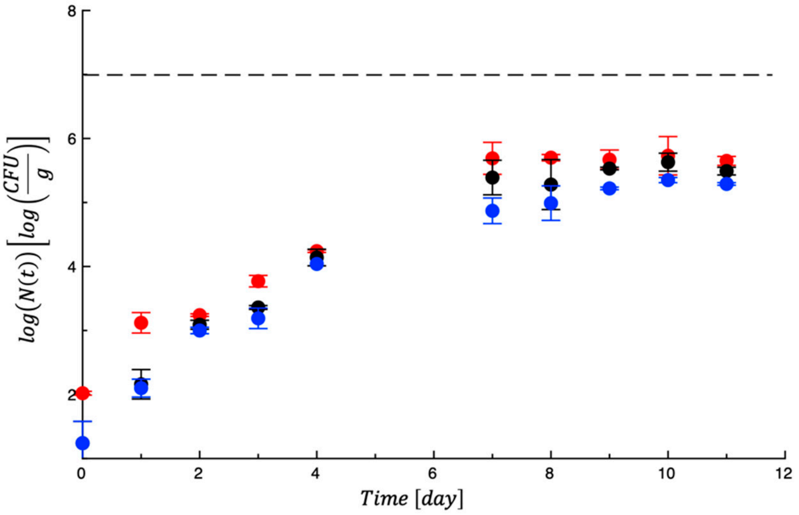

c)

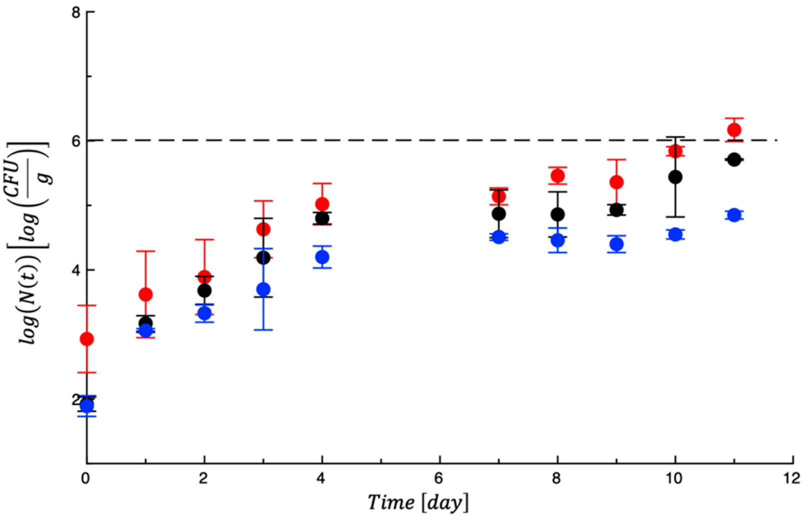

Figure S7: Evolution of mesophilic bacteria (a), lactic acid bacteria (b) and Enterobacteria (c) in turkey meat samples during storage. Data are means ± standard deviations. Red = CTN; Black = PET; Blu = PET-ZnO/Cu

a)

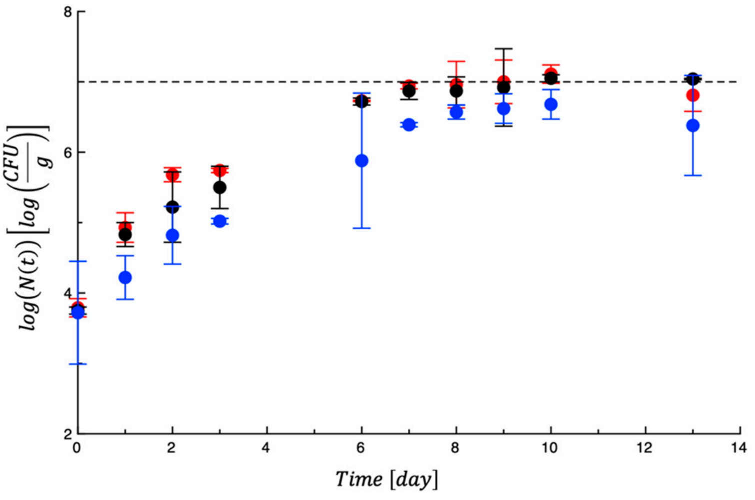

b)

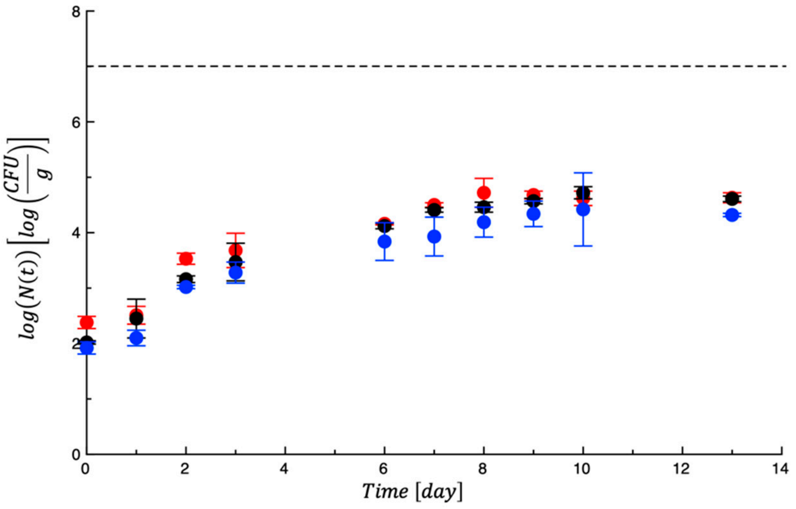

c)

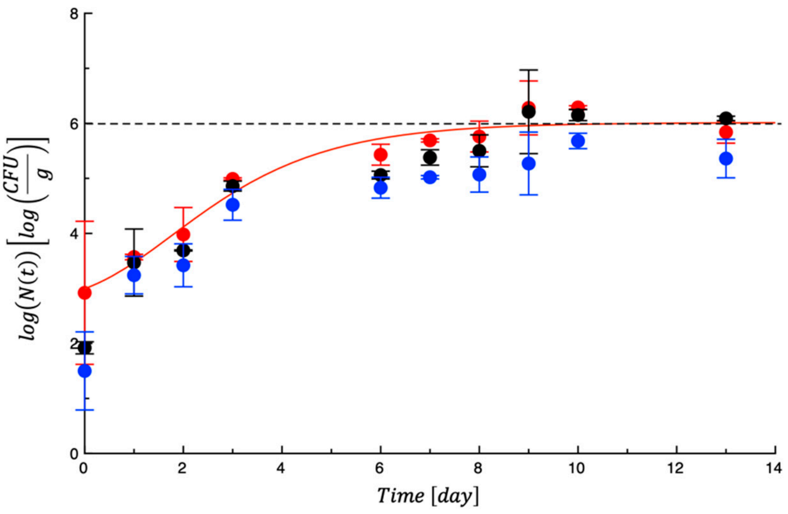

Supplement: Supplementary file 1 [file polymers-18-00751-s001.zip › polymers-4202269-supplementary.pdf]
